# Role of Frailty Index-Laboratory to predict COVID-19 mortality: a prospective study

**DOI:** 10.3389/fpubh.2025.1591767

**Published:** 2025-07-02

**Authors:** Elda De Vita, Nicola Veronese, Giacomo Guido, Luisa Frallonardo, Sergio Cotugno, Giorgia Manco Cesari, Marinella Cibelli, Alessandra Vigna, Davide Capruzzi, Monica Fiorella, Carmen Rita Santoro, Gaetano Brindicci, Francesco Di Gennaro, Annalisa Saracino

**Affiliations:** ^1^Clinic of Infectious Diseases, Department of Precision and Regenerative Medicine and Ionian Area - (DiMePRe-J), University of “Aldo Moro”, University of Bari “Aldo Moro”, Bari, Italy; ^2^Department of Health Promotion, Mother Child Care, Internal Medicine and Medical Specialties, University of Palermo, Palermo, Italy

**Keywords:** frailty, Frailty Index-Laboratory, aging, COVID-19, mortality

## Abstract

**Introduction:**

The COVID-19 pandemic has disproportionately impacted frail individuals, highlighting the urgent need for effective prognostic tools to improve patient outcomes. Early identification of at-risk individuals can optimize management and resource allocation, reducing mortality and morbidity. This study evaluates the Frailty Index-Laboratory (FI-LAB) as a predictor of mortality in COVID-19 patients.

**Methods:**

We included all COVID-19 patients admitted to the Clinic of Infectious Diseases of the “Azienda Ospedaliera Policlinico di Bari” from March 2020 to February 2024. FI-LAB scores were calculated using 37 laboratory parameters obtained within the first 4 days of hospitalization. Mortality data were collected up to 90 days post-admission. Cox regression analysis, adjusting for demographics, comorbidities, COVID-19 symptoms, and vaccination status, was employed to examine the relationship between FI-LAB scores and mortality.

**Results:**

One thousand, four hundred ninety-two patients were included in the study population, the mean age was 57.2 years (SD = 15.9), with 56.6% being male. Patients in the highest FI-LAB tertile (>0.432) exhibited a 17.10-fold higher risk of death compared to those in the lowest tertile (<0.135), same result has been shown in the intermediate FI-LAB scores (0.135–0.432) when compared to the lowest tertile. Additionally, each 0.10-point increase in FI-LAB was linked to a nearly twofold increase in mortality hazard (HR = 1.99, 95% CI 1.69–2.37, *p* < 0.0001).

**Conclusion:**

Frailty Index-Laboratory is a robust and practical tool for predicting mortality in hospitalized COVID-19 patients, aiding early identification of high-risk individuals. Implementing FI-LAB enhances patient management and resource allocation. Further studies are needed to confirm its effectiveness across diverse populations and healthcare settings.

## Introduction

The global emergency caused by COVID-19 pandemic has highlighted the differential impact of the virus on the population, particularly concerning frail individuals (1). During the early stages of the COVID-19 pandemic, high mortality rates were reported, likely due to the limited therapies and preparedness of public health systems to handle a novel disease with unprecedented epidemiological and pathophysiological characteristics (2). The synergy between COVID-19 and frailty is complex and multifactorial, involving inflammatory, immunological, and metabolic processes that accelerate the functional and cognitive decline of frail individuals, ultimately leading to death (3). Nowadays, with the significant implementation of vaccinations and therapies, we are experiencing a different phase of the COVID-19 pandemic (4). However, as frail patients are more vulnerable and often underrecognized, it is crucial to tailor therapeutic and prophylactic interventions by identifying them using increasingly manageable and applicable tools in every setting. Focusing solely on older patients usually affected by several comorbidities as risk or frail patients may be misleading, as some authors suggest (5). A more comprehensive assessment is necessary for a better understanding of frailty, similar to what the Frailty Index allows (6). In this context, the Frailty Index (FI) has emerged as a crucial indicator for understanding the impact of COVID-19, particularly in older people and individuals with preexisting health conditions. These populations present a greater likelihood of death and other unfavorable outcomes (7).

As regards, some authors recently promoted the use of a more accurate tool, the Frailty Index-Laboratory (FI-LAB), which uses laboratory data to evaluate frailty, offering a more objective approach than the traditional clinical parameter-based FI (8). The FI-LAB is dedicated to the creation and verification of instruments for assessing frailty, particularly in older patients, with the goal of enhancing patient outcome evaluation and customizing healthcare interventions (9). The FI-Lab’s components are derived from commonly measured hospital tests, simplifying its calculation and implementation for the early detection of frailty in clinical settings. This represents an evolution in frailty assessment by incorporating objective laboratory data alongside clinical parameters. It underscores the pivotal role of laboratory research in enhancing the understanding of frailty dynamics across various clinical contexts (9). Differently from a pure clinical assessment of frailty, the FI-LAB offers, indeed, automation advantages providing a novel perspective on frailty and improving predictive capability when integrated with clinical frailty indexes in hazard models (10). Being easily applicable in many healthcare contexts, this tool can be readily used becoming an ideal tool for detecting and screening frailty early on, potentially improving patient care and outcomes. Its adoption in clinical practice holds promises for improving the detection and management of frailty, thereby potentially enhancing patient care and quality of life (8). Nonetheless, the existing literature on the use of FI-LAB in patients affected by COVID-19 is limited and further research is needed to fully confirm the validity, accuracy, and reliability of FI-LAB in this specific population, characterized by a potential high risk of mortality (11, 12). Our rationale for applying the Lab-FI in this cohort, which included both older and younger adults, stems from the desire to explore whether frailty—as a marker of reduced physiological reserve—could serve as a predictor of adverse outcomes beyond the geriatric population. For this reason, the purpose of our study is to investigate the applicability and use of the FI-LAB as a predictive tool for estimating mortality in a large cohort of COVID-19 patients who have been admitted to our hospital.

## Materials and methods

### Study population

We conducted a prospective cohort study enrolling 1,492 patients, all patients aged 18 years or older were enrolled at the “Azienda Ospedaliera Policlinico di Bari” in Bari between March 7, 2020, and February 3, 2024. The diagnosis of SARS-CoV-2 infection was verified by RT-PCR upon finding SARS-CoV-2 nucleic acid on a nasopharyngeal swab.

A signed consent for all eligible subjects was acquired during hospitalization (retrospective data). The Local Ethical Committee of the Azienda Ospedaliera Policlinico di Bari approved the study (number 7280, 04/2022), performed in accordance with the ethical standards as laid down in the 1964 Declaration of Helsinki and its later amendments or comparable ethical standards.

A signed informed consent for all eligible subjects was acquired during hospitalization (retrospective data).

### Exposure: construction of the FI-LAB

During the first 4 days of hospitalization, 37 distinct laboratory data were gathered to build the FI-LAB. These values included blood counts, liver and renal function, pancreatic, blood glucose, and lipid profiles, serum electrolytes, coagulation parameters, inflammatory parameters, blood gas analysis parameters, serum vitamin D levels, and thyroid profiles. Supplementary Table 1 has a complete list of all the factors considered. If the subject’s values were normal, a value of 0 was assigned to each parameter; if they were abnormal, a value of 1. After that, we divided the total number of abnormalities by the total number of exams that were offered for a subject whose final score fell between 0 and 1; larger numbers indicated a greater number of abnormalities (13). Since no univocal cut-offs for FI-LAB exist, for the aims of this research, the FI-LAB was categorized into tertiles, a method already used in other similar works (14, 15).

### Outcome: mortality

Death certificates and the medical records’ accompanying documentation were used to record mortality. No participant was lost during follow-up, being able to record all the information about mortality for all the patients initially included.

### Covariates

Other than age and sex, we have included the following covariates, based on the literature about FI-LAB and mortality (16):

- Demographics: Nationality, smoking status;
- Presence of comorbidities (hypertension, hypercholesterolemia, diabetes mellitus, obesity, cancer) adjudicated using medical records, medical history, physical examination and previous laboratory data;
- COVID-19 symptomatology, according to the most common signs and symptoms of this disease;
- Vaccination status against SarsCoV2.

### Statistical analysis

Patients were divided according to their initial FI values using tertiles (cut-offs 0.135 and 0.432). For continuous variables, values were presented as absolute and relative frequencies (in percentage terms) or as means and standard deviations (SD) for each of the three groups. The Kolmogorov–Smirnov test was utilized to evaluate the normality of the continuous variables. The ANOVA test for independent samples was used to compare continuous variables between the three groups, and the Chi-Square test was used to analyze categorical variables, with Fisher’s correction used as needed. The Bonferroni’s adjustment was used. After checking the proportionality assumption, Cox regression was used to examine the relationship between the FI-LAB and mortality after adjusting for various variables.

Factors resulted statistically different between patients (*p*-value < 0.05) or linked with the outcome of interest (*p* < 0.10) were included in the multivariate analysis. Multicollinearity was assessed using the variance inflation factor, using a value of 2 as thresholds: however, none of the factors included in the multivariate analysis was excluded for this reason. The findings were then presented as 95% confidence intervals (95%CI) and the hazard ratio (HR). When a *p*-value was less than 0.05, we regarded the results as statistically significant. We classified this variable in tertiles since the FI-LAB lacked a clear cut-off. The FI-LAB score was then simulated as increasing by 0.10 points. Lastly, we conducted a study to see how well the FI-LAB predicted death when combined with age and gender. The results were reported as the area under the curve (AUC) and its 95% confidence interval (CI).

SPSS 26.0 for Windows (SPSS Inc., Chicago, Illinois) was used for all analyses. Every statistical test employed a two-tailed design, with a *p*-value of less than 0.05 considered statistically significant.

## Results

Our study includes 1,492 patients admitted with a diagnosis of COVID-19 disease in our hospital from 7 March 2020 to 3 February 2024. The patients aged a mean of 57.2 years (SD = 15.9, range: 21–93), mainly males (56.6%) and Italian (52.3%).

As shown in Table 1, age did not significantly differ across the three FI-LAB groups (*p* = 0.20). The male population was significantly more prevalent in those having an intermediate risk (FI 0.135–0.432) group compared to the other two groups (*p* = 0.005) as well as the prevalence of Italian patients (*p* < 0.0001). Comorbidities were significantly more prevalent in the FI-LAB 0.135–0.432 and FI > 0.432 groups compared to FI-LAB <0.135 (*p* < 0.0001). In particular, hypertension and hypercholesterolemia were more common in the FI-LAB 0.135–0.432 group (*p* < 0.0001) as well as diabetes mellitus prevalence (*p* < 0.0001), while obesity rates did not significantly differ among FI groups (*p* = 0.23).

**Table 1:** Descriptive characteristics of the participants included by frailty index.

Variable	FI-LAB <0.135 (*n* = 528)	FI-LAB 0.135–0.432 (*n* = 499)	FI-LAB >0.432 (*n* = 465)	*p*-value
Age	57.5 (15.5)	56.2 (15.4)	57.9 (16.8)	0.20
Males	53.2	57.9	57.8	0.005
Italian	35.4	67.9	54.8	<0.0001
Current smokers	2.5	1.8	3.7	0.26
Comorbidities	46.8	64.9	59.1	<0.0001
Hypertension	30.3	46.5	43.0	<0.0001
Hypercholesterolemia	6.8	19.8	9.5	<0.0001
Diabetes mellitus	9.3	18.2	18.1	<0.0001
Obesity	10.8	22.4	13.1	0.23
Cancer	21.0	17.6	23.4	0.39
COVID-19 symptomatology				
Dyspnoea	7.0	74.3	88.8	<0.0001
Anosmia	4.2	4.2	2.2	0.09
Dysgeusia	5.5	13.0	38.3	<0.0001
Fever	50.0	66.5	52.5	0.32
Cough	17.6	35.5	37.4	<0.0001
Gastrointestinal symptoms	8.5	18.4	45.8	<0.0001
Pneumonia	89.2	97.2	98.9	<0.0001
Use of oxygen during hospital stay	24.4	89.2	99.6	<0.0001
Vaccination against SarsCoV2	77.8	52.3	89.2	<0.0001

FI-LAB, Frailty Index-Laboratory.

COVID-19 symptomatology had, again, significant difference across FI-LAB groups with dyspnoea, dysgeusia, and cough being more prevalent in people with higher FI-LAB groups as well as gastrointestinal symptoms (*p* < 0.0001). Conversely, fever prevalence was similar across FI-LAB groups (*p* = 0.32) (Table 1). Pneumonia incidence was highest in the FI > 0.432 group as well as use of oxygen during hospital stay (*p* < 0.0001 for both comparisons) and, similarly, vaccination against SarsCoV2 was found higher in frailer patients (*p* < 0.0001).

**Figure 1:**
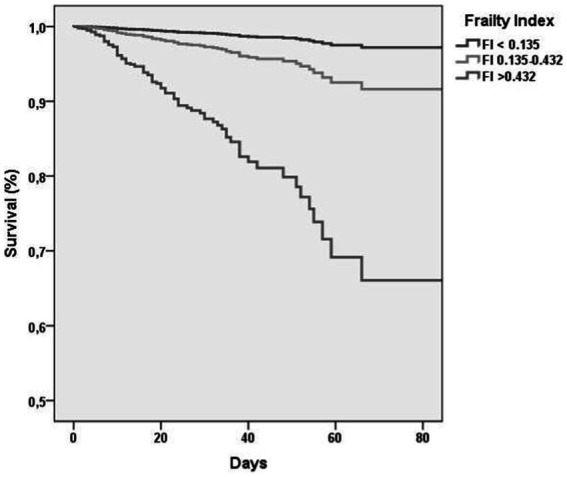
Survival curves, by frailty index lab values at baseline.

Table 2 shows association between FI-LAB at admission and mortality during follow-up. In particular, when adjusted for age and sex in the Model 1 of multivariate analysis, those with FI-LAB 0.135–0.432 had a 4.19-fold increased risk (95% CI 1.42–12.32, *p* = 0.009) of death compared to individuals with FI-LAB <0.135 as the reference group, in particular those with FI-LAB >0.432 resulted with a higher risk with an HR of 17.10 (95% CI 6.26–46.69, *p* < 0.0001) with the survival curve at 90 days markedly lower compared to the other groups (Figure 1). Similar trends were found after adjusted for additional covariates in Model 2 that includes 17 covariates about comorbidities, COVID-19 symptomatology, pneumonia findings and vaccination status, with FI-LAB 0.135–0.432 group not significantly associated with a 3.08-fold increased risk of death (95% CI 0.86–11.07, *p* = 0.09), while FI-LAB >0.432 maintained a substantial HR of 14.60 (95% CI 3.99–53.40, *p* < 0.0001). Moreover, modeling FI-LAB as a continuous variable, a 0.10-point increase in FI-LAB was associated with nearly doubled hazards in both Model 1 (HR 1.98, 95% CI 1.71–2.28, *p* < 0.0001) and Model 2 (HR 1.99, 95% CI 1.69–2.37, *p* < 0.0001).

**Table 2:** Association between frailty index and mortality.

Variable	Basic adjusted model^1^			Fully adjusted model^2^		
	HR	95% CI	*p*-value	HR	95% CI	*p*-value
FI-LAB <0.135	1			1		
FI-LAB 0.135–0.432	4.19	1.42–12.32	0.009	3.08	0.86–11.07	0.09
FI-LAB >0.432	17.10	6.26–46.69	<0.0001	14.60	3.99–53.40	<0.0001
Increase in 0.10 points	1.98	1.71–2.28	<0.0001	1.99	1.69–2.37	<0.0001

^1^Basic model was adjusted for age and sex; ^2^Fully adjusted model included age, sex, presence of hypertension, dyslipidaemia, diabetes mellitus, obesity, smoking status, cancer, dyspnoea, anosmia, dysgeusia, fever, cough, gastrointestinal symptoms, pneumonia, use of oxygen during the hospital stay, vaccination against SarsCoV2.

**Figure 2:**
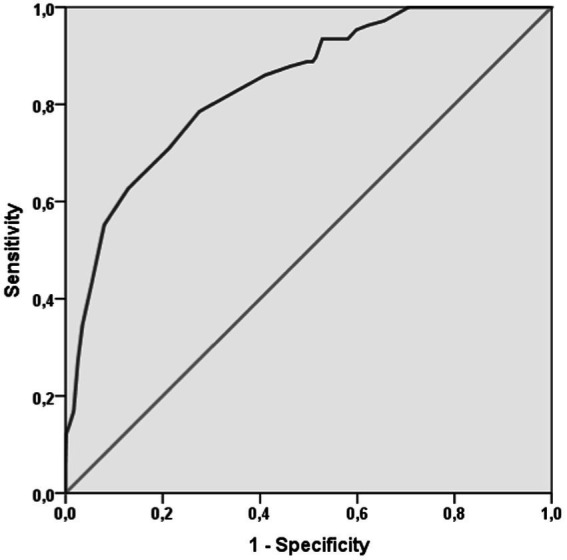
Accuracy of frailty index lab in predicting mortality.

Finally, as shown in Figure 2, FI-LAB had an elevated accuracy. FI-LAB at admission had an AUC = 0.839 (95%CI: 0.801–0.876, *p* < 0.0001). The value of FI-LAB of 0.135 had an elevated sensitivity (97.2%) and, as expected a low specificity (34.2%), while the cut-off of 0.432 had a good sensitivity (79%) and specificity (72%).

## Discussion

Our data indicate that the FI-LAB accurately predicts mortality risk in COVID-19 patients, with intermediate and high FI-LAB scores strongly associated with negative outcomes. This experience from a large cohort of over 1,400 COVID-19 patients reinforces the idea that an FI-LAB, created using standard bio-humoral tests commonly requested for clinical reasons, can enhance our ability to screen for frailty in both older and younger hospitalized patients.

The FI-LAB, relying on routine laboratory tests already part of standard clinical practice, is cost-effective, requiring no additional blood exams beyond the routine (17). It is easier to collect than scores based on clinical assessment, minimizing the need for additional patient evaluations and eliminating the need for subjective clinical judgment and detailed patient interaction (10). This efficiency makes FI-LAB a more practical tool for preliminary frailty screening and supports its widespread use in various healthcare settings.

In the literature so far only three studies have examined the FI-Lab’s role in COVID-19 among older adults (8, 11, 18). The last published study, conducted by Veronese et al. (8), differently from the previous ones, included only laboratory measures, excluding clinical parameters, and incorporating blood tests used in respiratory. According with the analysis each 0.10-point increase in FI-LAB corresponded to an 80% increase in mortality risk suggesting that factors commonly altered in COVID-19 may have enhanced the predictive accuracy of the FI-LAB tool compared to previous literature (8). These data were similar to those found in our work, in which an increase in 0.10 in the FI-LAB was associated with an increased risk of about 80%. Our work, based on a much larger sample, has led us to similar conclusions, revealing that patients with intermedium FI-LAB had a 4.19-fold increased risk of death compared to individuals with the lowest FI-LAB as the reference group, and individuals with the highest FI-LAB resulted with a higher risk with an HR of 17.10 with the survival curve at 90 days markedly lower compared to the other groups.

Furthermore, when compared to clinical frailty index scores, despite methodological differences, our reported effect size is comparable, introducing the FI-LAB as a highly accurate predictor of mortality, with an AUC of 0.83. This indicates its strong potential for identifying frail patients at higher risk of mortality.

From our analysis emerged the relatively young median age (around 57 years) of our population when compared to the national varying between a mean age of 63.9 years among hospitalized COVID-19 patients during the early phase of the pandemic (19) and the median age of 70 years reported by a comprehensive analysis of the Italian National Institute of Health (Istituto Superiore di Sanità, ISS) (20). This reinforces the validity of the FI-LAB score even in younger populations, in contrast to previous studies that have validated this score primarily in older adults, revealing that FI-LAB is more accurate among older people than in younger patients (21). On the contrary, as shown in Figure 2, after adjusting for sex and age, FI-LAB remained highly specific and accurate revealing a strong correlation between FI and death risk among younger patients. For this reason, although the FI-LAB score is typically used for older adults, it has also shown potential in assessing vulnerability and predicting adverse outcomes in younger patients with chronic conditions. This underscores the importance of using the FI-LAB as a comprehensive tool for early intervention and tailored treatment plans in younger patient groups, potentially enhancing their long-term health outcomes.

Regarding potential gender differences, we discovered a higher presence of male subjects in the intermedium FI-LAB, with no gender effect on mortality, as already stated by other studies in literature (16). In particular, a meta-analysis conducted by Snapp et al. (16) found that three studies reported women scored lower on the FI-LAB than men, while two studies found the opposite, and five studies found no sex difference (22). However, when compared to alternative frailty measures, such as the Clinical Frailty Scale (CFS), results diverge showing gender differences among COVID-19 patients (23).

In this regard, a prospective multicenter study using the Clinical Frailty Scale (CFS) on over 3,000 aged individuals showed that male COVID-19 patients tended to have more severe outcomes and higher mortality rates compared to female patients. This disparity persisted even after adjusting for age and comorbidities (22). This phenomenon reflects a wider problem where differing operational definitions of frailty and the variety of data collected can lead to inconsistent epidemiological and clinical conclusions (23). If some studies have found that men tend to show higher levels of frailty at a younger age compared to women, potentially due to differences in life expectancy, health behaviors, and biological factors conversely, on the other hand women often exhibit a higher prevalence of frailty in old age, possibly due to a longer life expectancy (24). This emphasizes the importance of selecting appropriate frailty measures that can apply to younger patients as well and considering gender differences in clinical assessments and research. As suggested in other studies (8), expanding diagnostics to include in the FI-LAB hormonal markers to identify gender differences could help to better discriminate risk classes.

In our cohort, from a clinical perspective, moderate and severe frailty, as indicated by FI-LAB, were more frequent in diabetic and hypertensive individuals, as well as in patients with hypercholesterolemia. As already demonstrated in the literature, frailty in COVID-19 patients is exacerbated in those with hypertension and diabetes, leading to higher risks of severe outcomes and mortality (25–27). Furthermore, COVID-19 in itself increases the frailty index among older individuals with comorbidity, exacerbating pre-existing vulnerabilities and leading to poorer health outcomes accelerating the decline in physiological reserves (28).

It is also noteworthy that in our analysis, in line with findings in the literature, COVID-19 symptomatology was more prevalent in higher FI groups, in particular cough, dyspnea, and gastrointestinal (GI) symptoms (29). This can be explained by the evidence that frail individuals have reduced gut microbiota diversity leading to worse outcomes of COVID-19 (30). Vaccination against SARS-CoV2 has been found with a higher rate in the higher FI group probably due to a weakened immune response to vaccinations, characterized by reduced antibody production and diminished effectiveness in mounting protective immunity common in frail individuals (4, 31). However, higher vaccination rate observed among frailer patients in our cohort likely reflect the national vaccination policies in effect during 2021 as in the early phases of the COVID-19 vaccine rollout, priority access was granted to older adults, individuals with multiple comorbidities, and other clinically vulnerable populations. Age, male sex, and seronegative status at baseline following vaccination in frail patients are particularly associated with lower levels of total anti-SARS-CoV-2 receptor-binding domain (RBD) antibodies (32), which increases the likelihood of severe COVID-19 (4). Furthermore, despite the three vaccination doses have the potential to maintain high antibody titers, however, within the first 6 months the antibody response is generally lower and rapidly reducing in fragile patients as compared to healthy people (33).

The COVID-19 pandemic has posed an unprecedented challenge to global public health. Beyond its acute manifestation, the virus has a potential impact on health and quality of life, even after clinical recovery from the infection (34). Recently, various factors, including age, anemia, diabetes, hypertension, and obesity, have been correlated with mortality predictions in COVID-19 patients (35). However, it has been observed that these somatic disorders are not sufficient to fully predict unfavorable outcomes in COVID-19 patients. Therefore, the identification of new prognostic risk indicators is necessary to adequately identify and stratify patients (13). As regards, in clinical practice, the FI-LAB may serve not only as an indicator of underlying frailty but also as a marker of overall physiological reserve and resilience in the face of acute illness. With a potential role in clinical risk stratification, FI-LAB could be a high accurate score to predict the mortality in patients affected by COVID-19 (8).

Our conclusions must be considered with limitations. Firstly, female participants were underrepresented in the laboratory tests, potentially introducing a gender bias, as also noted in other studies suggesting the inclusion of hormones in the FI-LAB. Additionally, the heterogeneity of the population is notable, as data has been collected both before and after vaccination campaigns and we have no data on reinfection rate. Furthermore, the clinical presentation and outcomes of COVID-19 evolved significantly across different pandemic waves, and no statistical adjustment was made for the period of hospital admission, which we acknowledge as a limitation disease severity and patient management. Lastly, the study focused exclusively on patients with COVID-19, limiting the generalizability of the results to frailty in patients without the virus. Notably, some of the 95% confidence intervals reported in our analysis are relatively wide, likely reflecting a limited number of events in certain FI-LAB categories. This reduced precision may affect the robustness of specific estimates and should be considered when interpreting the results. Future studies with larger cohorts or a more balanced distribution of events could help enhance the reliability of these associations.

In summary, our research indicates that mortality among frail adults hospitalized with COVID-19 can be predicted using a straightforward indicator derived from standard laboratory testing. Our results suggest that the FI-LAB could be a practical and reliable screening tool for rapidly assessing outcomes in frail patients, easily applicable in clinical settings.

## Data Availability

The raw data supporting the conclusions of this article will be made available by the authors, without undue reservation.
