# Supplementary material for: Role of Frailty Index-Laboratory to predict COVID-19 mortality: a prospective study
Source: Front Public Health. 2025 Jul 2;13:1591767. doi: 10.3389/fpubh.2025.1591767 (PMC12263403; doi:10.3389/fpubh.2025.1591767)
Supplement: Supplementary file 1 [file Data_Sheet_1.PDF]

Supplementary Table 1. Laboratory variables for frailty index

	Item	No frailty (Normal)*	+1 Frailty risk (Abnormal)
Routine blood test			
1	Hb (g/L)	115–150 (F)	<110 or >150 (F)
		130–175 (M)	<130 or >175 (M)
2	PLT (*10^9/L)	101–320	<101 or >320
3	WBC (*10^9/L)	3.5–9.5	<3.5 or >9.5
4	NEUT (*10^9/L)	1.8–6.3	<1.8 or >6.3
5	LYMPH (*10^9/L)	1.1–3.2	<1.1 or >3.2
Hepatic Function			
6	TBil (umol/L)	5.0–26.0	<5.0 or >26.0
7	DBil (umol/L)	≤8	＞8
8	IDBil (umol/L)	≤20	＞20
9	ALT (IU/L)	≤40 (F)	＞40 (F)
		≤50(M)	＞50(M)
10	AST (IU/L)	≤35 (F)	＞35 (F)
		≤40(M)	＞40(M)
11	ALB (g/L)	32–55	<32 or >55
12	ALP (IU/L)	35–135 (F)	<35 or >135 (F)
		45–125 (M)	<45 or >125 (M)

13	GGT (IU/L)	7-45 (F)	＜7 or ＞45 (F)
		10-60 (M)	＜10 or ＞60 (M)
14	CK (IU/L)	40–200 (F)	<40 or >200 (F)
		50–310 (M)	<50 or >310 (M)
15	LDH (IU/L)	120–250	<120 or >250
Fast Blood Glucose			
16	GLU (mmol/L)	3.9–6.11	<3.9 or >6.11
Renal Function			
17	CREA (umol/L)	41–81 (F)	<41 or >81 (F)
		57-111(M)	<57 or >111 (M)
Blood Lipid			
18	TG (mmol/L)	≤2.3	>2.3
19	CHOL (mmol/L)	≤5.6	>5.6
20	HDL-C (mmol/L)	≥1.15(F)	＜1.15(F)
		≥0.9(M)	＜0.9(M)
21	LDL-C (mmol/L)	≤4.11	＞4.11
Blood Electrolyte			
22	NA (mmol/L)	137.0–147.0	<137.0 or >147.0
23	K (mmol/L)	3.5–5.3	<3.5 or >5.3
Blood Coagulation			

24	INR	0.80–1.30	<0.80 or >1.30
25	Fib (g/L)	2.0–4.0	<2.0 or >4.0
26	D-dimer	<150	>150
Blood Respiratory			
27	pO2 (mmHg)	>60	<60
28	Ph	7.35-7.45	<7.35 or >7.45
29	PCO2 (mmHg)	35-45	<35 or >45
30	Lactates (mmol/L)	0.5 - 2.2	<0.5 or >2.2
31	P/F ratio	>150	<150
Inflammation			
32	CRP (mg/L)	<5	>5
33	Procalcitonin (pg/ml)	<0.05	>0.05
Hormones			
34	Vitamin D (nmol/l)	50-150	<50 or >150
35	TSH (mIU/L)	0.4-4.0	<0.4 or >4.0
Pancreas			
36	Amilases (UI/L)	<150	>150
37	Lipases (UI/L)	<150	>150
Abbreviations: F, female; M, male; RBC, red blood cell; Hb, hemoglobin; HCT, hematocrit; MCV, mean corpuscular volume; MCH, mean corpuscular hemoglobin; MCHC, mean corpuscular hemoglobin concentration; RDW-CV, red cell distribution width-coefficient of variation; RDW-SD, red cell distribution width-standard deviation; PLT, platelets; WBC, white blood cell; NEUT, neutrophil; LYMPH, lymphocyte;			

MONO, monocyte; EO, eosinophil; BASO, basophil; TBil, total bilirubin; DBil, direct bilirubin; IDBil, indirect bilirubin; ALT, alanine transaminase; AST, aspartate aminotransferase; TP, total protein; ALB, albumin; ALP, alkaline phosphatase; GGT, gamma-glutamyl transpeptidase; CK, creatine kinase; LDH, lactate dehydrogenase; GLU, glucose; UREA, urea; CREA, creatinine; URIC, uric acid; TG, triglyceride; CHOL, cholesterol; HDLC, high-density lipoprotein cholesterol-C; LDL-C, low-density lipoprotein cholesterol-C; NA, sodium; K, potassium; CL, chlorinum; MG, magnesium; CA, calcium; P, phosphorus; PT, prothrombin time; INR, international normalized ratio; APTT, activated partial thromboplastin time; Fib, fibrinogen.
